# Differential resting-state functional connectivity of anterior and posterior cerebellar divisions with canonical networks in PTSD

**DOI:** 10.1016/j.xjmad.2026.100209

**Published:** 2026-10-20

**Authors:** LeeAnne Tunstall, Nick Steele, Ahmed Hussain, Courtney Russell, Jenna Beakas, Ashley A. Huggins, Jennifer S. Stevens, Kerry Ressler, Tanja Jovanovic, Sanne J.H. van Rooij, Negar Fani, Judith K. Daniels, Anika Sierk, Antje Manthey, Henrik Walter, Daniel W. Grupe, Jack B. Nitschke, Richard J. Davidson, Lee A. Baugh, Raluca M. Simons, Jeffrey S. Simons, Vincent A. Magnotta, Kelene A. Fercho, Ye Zhu, Gen Li, Li Wang, Rajendra A. Morey, Delin Sun

**Affiliations:** a Brain Imaging and Analysis Center, Duke University, Durham, NC, USA; b Department of Veteran Affairs, Mid-Atlantic Mental Illness Research, Education and Clinical Center, Durham, NC, USA; c Department of Psychiatry and Behavioral Sciences, School of Medicine, Duke University, Durham, NC, USA; d Department of Psychology, University of Arizona, Tucson, AZ, USA; e Department of Psychiatry and Behavioral Sciences, Emory University School of Medicine, Atlanta, GA, USA; f Division of Depression and Anxiety Disorders, McLean Hospital, Belmont, MA, USA; g Department of Psychiatry, Harvard Medical School, Boston, MA, USA; h Department of Psychiatry and Behavioral Neuroscience, Wayne State University School of Medicine, Detroit, MI, USA; i Department of Clinical Psychology, University of Groningen, Groningen, the Netherlands; j TraumaCentrum Beilen, GGZ Drenthe, the Netherlands; k University Medical Centre Charité, Berlin, Germany; l Center for Healthy Minds, University of Wisconsin-Madison, Madison, WI, USA; m Department of Psychiatry, University of Wisconsin-Madison, Madison, WI, USA; n Department of Psychology, University of Wisconsin-Madison, Madison, WI, USA; o Division of Basic Biomedical Sciences, Sanford School of Medicine, University of South Dakota, Vermillion, SD, USA; p Center for Brain and Behavior Research, University of South Dakota, Vermillion, SD, USA; q Sioux Falls VA Health Care System, Sioux Falls, SD, USA; r Department of Psychology, University of South Dakota, Vermillion, SD, USA; s Departments of Radiology, Psychiatry, and Biomedical Engineering, University of Iowa, Iowa City, IA, USA; t Federal Aviation Administration, Oklahoma City, OK, USA; u Laboratory for Traumatic Stress Studies, Chinese Academy of Sciences Key Laboratory of Mental Health, Institute of Psychology, Chinese Academy of Sciences, Beijing, China; v Department of Psychology, University of Chinese Academy of Sciences, Beijing, China

**Keywords:** PTSD, Cerebellum, Connectivity, Cortical Networks

## Abstract

**Background:**

Posttraumatic stress disorder (PTSD) is a complex psychiatric condition that may develop following exposure to a singular or series of traumatic events producing disruptions in cognitive and affective processes. Although the cerebellum is traditionally linked to voluntary movement coordination and balance, recent work highlights posterior cerebellar regions are involved in affective and cognitive functions. Our goal was to investigate functional connectivity between the cerebellum and canonical cortical resting-state networks in PTSD, with a particular focus on the posterior cerebellum based on previously reported structural changes associated with PTSD on the posterior cerebellum.

**Methods:**

A total of 313 participants ($M_{\mathrm{age}}$= 35, 218 females) were comprised of two groups; 119 PTSD patients and 194 healthy controls from eight study sites within the ENIGMA-PTSD working group. The cerebellum was segmented using *ACAPULCO* into posterior and anterior divisions. Resting-state fMRI preprocessed with *HALFpipe* was used to assess the functional connectivity of the anterior and posterior divisions of the cerebellum with major canonical cortical networks.

**Results:**

Compared to the non-PTSD control group, the PTSD group had greater connectivity of the posterior cerebellum with somatomotor (t = 4.32, p = 0.040) and default mode (t = 4.41, p = 0.040) networks. Correspondingly, PTSD symptom severity was linked to greater connectivity differentially affecting the posterior relative to the anterior cerebellum with major cortical networks. PTSD symptom severity was positively associated with greater posterior cerebellum connectivity with the default mode (t = 4.32, p_fdr_=0.020), somatomotor (t = 3.57, p_fdr_=0.040), limbic (t = 3.74, p_fdr_=0.036), salience (t = 3.61, p_fdr_=0.040) and central executive network (t = 3.59, p_fdr_=0.040).

**Conclusion:**

PTSD symptom severity is associated with altered connectivity of the posterior cerebellum with default mode, salience and central executive networks. Cortical connections with the posterior cerebellum, an important socioemotional hub, are involved in PTSD pathology.

## Introduction

Posttraumatic stress disorder (PTSD) is a complex psychiatric condition that may occur because of experiencing or witnessing a singular or series of traumatic events such as combat, physical assault, sexual assault, natural disasters, or abuse. PTSD is characterized clinically by intrusive re-experiencing symptoms, avoidance, negative changes in cognition and mood, and hyperarousal (American Psychiatric Association [APA], 2022). The medial temporal regions, such as the hippocampus and amygdala, as well as ventral and medial prefrontal regions, have been widely implicated in the clinical features of PTSD [1]. By contrast, the cerebellum has received scant attention for its role in PTSD. Although the cerebellum is primarily recognized for its role in voluntary movement coordination and balance [2], studies have established that the cerebellum’s role extends far beyond motor control, including encoding emotional information, emotional learning, and the regulation of emotional states in relation to motor, cognitive, and social behaviors [3]. A resting state functional connectivity (rsFC) study has also indicated that the cerebellum is functionally interconnected with non-motor cortices that communicate with large-scale brain networks [4]. Therefore, explaining PTSD associated behaviors and symptoms necessarily involves investigating cerebellar connectivity with the cortex and canonical brain networks.

The cerebellum, located in the hindbrain, is divided into left and right hemispheres, which can be further split into three lobes. The flocculonodular lobe (archicerebellum) receives fibers input from the vestibular system, which plays an important role in balance and spatial orientation. The anterior lobe (paleocerebellum) receives direct fibers input from the spinal cord. The posterior lobe (neocerebellum), which is the largest of the three lobes, is densely interconnected with several areas of the cerebral cortex involved with cognitive function, social and affective processing, sensory evaluation, and motor planning [5], [6]. Resting-state functional connectivity reveals that the anterior cerebellum (paleocerebellum) has robust connections to motor, premotor, and somatosensory cortices, and the posterior (neocerebellar) subregions connect to the dorsolateral prefrontal (dlPFC) and inferior posterior parietal cortex (IPPC) to support executive functioning and cortical language processing circuits [7], [8]. The posterior cerebellum consistently shows strong connectivity with the dorsal medial prefrontal cortex (dmPFC) and the right temporo-parietal junction (TPJ), which support social cognition and mentalizing [9].

Cerebellar volume and subregion volumes are markedly lower in PTSD ( [10], [11], [12], suggesting the cerebellum may play a role in the pathophysiology of PTSD. Interestingly, volume measures for the evolutionarily modern posterior cerebellum, which is involved in social cognition and fear processing, are most affected in PTSD, whereas the evolutionarily older archicerebellum, which is involved in movement and coordination, appears largely preserved. Beyond these structural findings, altered cortico-cerebellar connectivity has been reported in PTSD [13], [14]. Lower cerebellar volume in select subregions provided the present impetus to investigate functional differences that might be associated with PTSD. Specifically, functional diversity within the cerebellum and its connectivity throughout the brain may implicate select cortico-cerebellar circuits in PTSD symptoms. Although regions such as the hippocampus, amygdala, medial prefrontal cortex, and anterior cingulate cortex have been extensively examined in PTSD, their dense interconnection with the cerebellum has been largely overlooked in the PTSD literature**.** This represents an important gap, as the posterior cerebellum has extensive anatomical and functional connections with prefrontal, limbic, and other cortical and subcortical regions that are strongly implicated in PTSD [15], [16], [17], [18]. The cerebellum’s widespread connectivity with stress-related regions (such as amygdala, hippocampus and periaqueductal gray) make it vulnerable to traumatic stress, potentially leading to the development of PTSD symptoms by disrupting typical brain-mediated stress responses via cerebro-cerebellar circuits. Carletto, Borsato [18], [19], [20] elucidates the extensive theoretical relevance of the posterior cerebellum by exploring its connectivity with the rostral cingulate cortex, which in turn communicates with the fastigial nucleus of the cerebellar vermis to modulate threat response behaviors such as threat orienting, vigilance, arousal, wakefulness, freezing, motivation, and other related behaviors. Furthermore, emerging evidence implicates the cerebellum in fear learning and memory [10], [21], [22], processes that are particularly relevant to PTSD given alterations in threat detection and processing are associated with the disorder [23], [24]. Thus, rather than prioritizing the posterior cerebellum over established PTSD-related regions, examining their interconnectedness may provide insight into an understudied component of the broader neural circuitry associated with PTSD.

Notably, PTSD has previously been linked to disruptions in intrinsic functional neural networks. Within this framework, disruptions to the default mode network (DMN) are correlated to some PTSD symptoms that include difficulty regulating emotions and intrusive thoughts [25]. The default mode network (DMN) is primarily involved in internally directed processes, including self-referential and reflective activity, and typically shows decreased activity during externally focused tasks. However, its engagement is context-dependent and can increase when external stimuli require internally oriented processing, such as in social contexts or solving problems. Davey et al., [26]. The DMN does not function in isolation; it interacts with other large-scale networks, which affects how the brain deals with emotional and cognitive demands. The central executive network (CEN), also known as the frontoparietal network, is a task-positive network that displays an inverse or reciprocal relationship with the DMN [27]. Thus, engaging the CEN will inhibit the DMN, whereas disengaging the CEN will activate the DMN [28]. In mediating this relationship, the salience network (SN) acts as a switch that directs contrasting network functions [29]. When presented with salient stimuli, the SN will quiet the DMN and switch to the CEN to prioritize essential information and emotional responses [30]. Dyssynchrony between these three networks is important to PTSD symptom presentation. Re-experiencing and intrusive trauma memories, which reflect internally focused processes, are associated with DMN hyperactivity [31]. Importantly, engaging the CEN may reduce self-referential thinking by quieting the DMN [27] and effectively abating re-experiencing symptoms [32]. There is limited evidence of altered cerebellum connectivity to the DMN [13] and the CEN in PTSD [33], however these studies have focused on connectivity of the whole cerebellum as a single region, but the integrity of functional networks that preferentially link specific posterior cerebellar or archicerebellar substructures to the cortex has not yet been investigated in PTSD.

The overall goal of our study was to examine resting-state functional connectivity to both the anterior and posterior cerebellum with canonical cortical networks in PTSD, with an a priori focus on the posterior cerebellum. Anterior cerebellar regions were included as a comparison segment to determine if PTSD-related differences were preferentially associated or more prominent within the posterior region. We applied the mega-analytic approach of the ENIGMA-PTSD consortium workgroup. Combining individual participant data from multiple sites offers greater statistical power, enhanced generalizability, and more precise links between symptoms and network connectivity [34]. Based on converging evidence from cortico-cerebellar network architecture and posterior cerebellar atrophy in PTSD, we hypothesized (1) greater posterior cerebellum-to-DMN connectivity in PTSD, (2) greater posterior cerebellum-to-CEN connectivity in PTSD, and (3) greater posterior cerebellum-to-SN connectivity in PTSD based on previously reported structural alteration in these regions. Moreover, we hypothesized that greater PTSD severity would be associated with greater cortico-cerebellar connectivity. Given that there is high comorbidity between PTSD and Major Depressive Disorder (MDD) [35], there will be a supplementary analysis to examine whether MDD diagnosis contributes to functional connectivity differences.

## Methods and materials

### Participants

There were 313 participants (218 female, 95 male) evaluated for current and lifetime PTSD (119 PTSD, 194 trauma-exposed controls) and Major Depressive Disorder (MDD). 72 PTSD participants and 32 controls met criteria for MDD. This reduced sample size reflects the inclusion criteria used for conducting our present analyses out of many sites involved in ENIGMA-PTSD, which required sufficient cerebellar coverage in functional imaging data. All study procedures were approved by local institutional review boards (IRBs), and participants provided written informed consent. The present secondary data analyses were granted exempt status by the Duke University Health System IRB. Table 1 provides clinical and demographic details of the study sample.

**Table 1:** Clinical Characteristics.

	**Controls (n = 194)**	**PTSD group (n = 119)**	**Statistic**	**df**	**p-value**
Sex (Female), n (%)	127 (65.5%)	91 (76.5%)	χ²= 3.72	1	0.054
Age (years)	35.82 ± 12.76 (18.00–67.00)	35.61 ± 11.32 (18.00–61.00)	t = 0.15	272.39	0.88
PTSD Severity (0−1)	0.17 ± 0.13 (0.00–0.59)	0.54 ± 0.13 (0.26–0.94)	t = 24.11	242.81	< 0.00001
Cluster B (0−1)	0.17 ± 0.20 (0.00–0.70)	0.50 ± 0.17 (0.07–0.95)	t = 8.59	90.49	< 0.00001
Cluster C (0−1)	0.24 ± 0.21 (0.00–0.75)	0.62 ± 0.18 (0.25–1.00)	t = 5.79	17.91	< 0.00001
Cluster D (0−1)	0.10 ± 0.09 (0.00–0.25)	0.43 ± 0.19 (0.07–0.89)	t = 8.59	42.93	< 0.00001
Cluster E (0−1)	0.24 ± 0.19 (0.00–0.67)	0.57 ± 0.13 (0.29–0.83)	t = 9.89	80.64	< 0.00001

PTSD: Posttraumatic Stress Disorder, Diagnostic status was determined using CAPS4: Clinician Administered PTSD Scale for DSM-IV, Clinician-Administered PTSD scale for DSM-5 (CAPS-5), PCL: PTSD Checklist for DSM-5, DSM-5 PTSD criteria: Cluster B: Intrusion symptoms; Cluster C: Avoidance symptoms; Cluster D: Negative alterations in cognition and mood; Cluster E: Alterations in arousal and reactivity.

#### Clinical Characteristics

PTSD diagnosis and symptom severity were investigated using clinician-administered interviews and validated self-report measures. There were differences in measures employed at some sites due to contrasting protocols to gather data at each site. The Clinician-Administered PTSD scale for DSM-IV (CAPS-IV), the Clinician-Administered PTSD scale for DSM-5 (CAPS-5), the PTSD checklist for DSM-5 (PCL-5) and the PTSD Symptom Scale-Self Report (PSS-SR) were used. At two sites, PTSD classification required a PCL-5 score ⩾ 32 for at least one month prior to the index trauma or if they met criteria for current PTSD. Blevins et al., [36], [37], [38] One site used the Clinician-Administered PTSD Scale for DSM-IV (CAPS-IV) for diagnosis and determined symptom severity using either the MPSS-SR or CAPS-IV. To harmonize PTSD symptom severity scores across different scales, raw scores were transformed to a 0–1 range using min-max normalization (e.g.[11], [39], [40]). DSM symptom cluster severity scores were also harmonized for intrusion (Cluster B), avoidance (Cluster C), negative alterations in mood and cognition (Cluster D), and alterations in arousal and reactivity. This was done by subtracting the minimum attainable score from the participant’s scores then dividing by the maximum attainable score minus the minimum attainable score resulting in that 0–1 severity range. {severity ∈ R∣0 < x < 1}. Depression diagnosis and severity were also evaluated using the Patient-Reported Outcomes Measurement Information System Depression Short Form (PROMIS DEP SF) [41], Center for Epidemiologic Studies Depression Scale (CES-D) [42], or Beck Depression Inventory (BDI) across various sites.

### Neuroimaging data acquisition & processing

**Functional Image Processing**: Functional MRI data (fMRI) were preprocessed using ENIGMA HALFpipe [43], a standardized pipeline that ensures reproducible fMRI analysis across study sites. The comprehensive preprocessing protocol included spatial smoothing with a 6.0 mm FWHM Gaussian kernel, slice timing correction, grand mean scaling and temporal filtering. Denoising fMRI data involved using *aCompCor* to generate principal components, (derivatives of) motion parameters (squared), white matter signal, CSF signal, global signal. These confound time series were regressed from fMRI time series data [44], [45]*.* Rigorous quality control criteria were enforced to exclude participants with poor skull stripping, segmentation problems, excessive head motion (>30% of frames with framewise displacement >0.5 mm), low temporal signal-to-noise ratio (tSNR outside the 25%-75% distribution range), completion errors, or registration problems.

**Cerebellar Parcellation and Spatial Transformation**: Cerebellar segmentation was performed using ACAPULCO (automatic cerebellum anatomical parcellation using U-Net with locally constrained optimization; [46]), which generated 28 cerebellar regions for each participant from their T1-weighted anatomical images. To align these anatomical regions with normalized functional data, the native space parcellations were transformed to MNI152NLin2009cAsym space using Advanced Normalization Tools (ANTs; antsApplyTransforms). This step applied the participant-specific T1w-to-MNI transformation matrices generated during preprocessing. A 2-mm MNI152NLin2009cAsym template served as the reference image to standardize the voxel resolution and spatial grid of the output parcellations. Nearest-neighbor interpolation was used to preserve categorical region labels. The vermal and flocculonodular subregions were excluded (Vermis VI, Vermis VII, Vermis VIII, Vermis IX, Vermis X, and X), and our analysis focused on left and right anterior (Lobules I–V) and posterior (Lobule VI, Crus I, Crus II, and Lobules VIIB, VIIIA, VIIIB, and IX) divisions. A key is provided in the supplementary materials providing the labels of each region.

**Resting-State Functional Connectivity**: Following spatial normalization, BOLD time series were extracted from the preprocessed resting-state images for 400 cortical regions defined by the Schaefer 400 atlas [47], which provided functionally homogeneous cortical regions organized according to the Yeo 17-network parcellation. This parcellation approach ensured high spatial resolution while maintaining neurobiologically meaningful network organization for functional connectivity analyses. Concurrently, time series for each of the 20 cerebellar regions were extracted using the transformed ACAPULCO parcellations. For all 420 cortical and cerebellar regions, the mean BOLD signal across voxels was computed to generate the regional time series. Functional connectivity was computed as the Pearson correlation coefficient between the time series of each of the 20 cerebellar parcels and each of the 400 cortical regions, yielding a 20 × 400 connectivity matrix. Finally, a Fisher *z*-transformation was applied to the connectivity matrices to stabilize the variance of the correlation coefficients, ensuring the connectivity measures were approximately normally distributed for downstream statistical analyses.

**Statistical Analysis:** Linear mixed-effects models implemented in the Image-Based Meta- and Mega-Analysis (IBMMA) toolkit [40] were used to examine the effects of PTSD diagnosis and symptom severity on functional connectivity. PTSD diagnosis (or overall symptom severity), age, age², and sex were modeled as fixed effects, with age² included to account for nonlinear effects of age. The models below in Table 2 provides the specifications for the primary and supplementary analyses. Study site was modeled as a random intercept to account for site-specific variance. MDD diagnosis was included as an additional fixed covariate in a supplementary analysis to account for the potential confounding influence of comorbid depression.Correction for multiple comparisons was applied across all cerebellar-to-cortical functional connections using the false discovery rate (FDR) method (q < 0.05, two-tailed). Cortical-to-cortical connections were not included in the analysis. Effect sizes (Cohen's d) and 95% confidence intervals (CIs) were reported for all statistically significant findings.

**Table 2:** Linear Mixed-Effects Model Specifications for PTSD Diagnosis and Symptom Severity.

FC = functional connectivity; PTSD = posttraumatic stress disorder; MDD = major depressive disorder. PTSD_Perc represents overall PTSD symptom severity. Age² represents the squared age term

**Table 3:** Cortical Cerebellar Network Connections in PTSD diagnosis.

DMN: Default Mode Network, SOM: Somatomotor Network, RH: Right Hemisphere, LH: Left Hemisphere, PFC: Prefrontal Cortex, AUD: Auditory

## Results

The analysis of functional connectivity between whole cortical networks and two cerebellar divisions (anterior and posterior) revealed group differences in PTSD compared to trauma-exposed controls particularly involving the default mode and somatomotor networks. Specifically, PTSD diagnosis was linked to a greater number of significant cortico-cerebellar functional connections of Lobules IV and V (anterior cerebellum) with the default mode network (Lobule IV: t = 4.41, q = 0.040), (Lobule V: t = 4.32, q = 0.040) (see Table 3). In addition, multiple significant functional connections of posterior cerebellar nodes, including lobules VIIb, VIIIb, Crus I, and II showed greater connectivity with the default mode, somatomotor A, and somatomotor B networks (t = 3.28–4.59, all FDR-corrected q ≤ 0.05). Supplementary materials provide a showcasing of the t-statistic for each connection. (e.g. Table S6)

Similar to PTSD diagnosis, PTSD severity was linked to greater connectivity of the posterior cerebellum with multiple networks, specifically the default mode, somatomotor, limbic, salience, and central executive. The anterior cerebellum also had greater connectivity with the default mode, salience, and central executive networks for the PTSD severity. Fig. 1 shows significant connections between anterior and posterior cerebellar regions with whole cortical networks for PTSD severity. Next, we tested our hypothesis that the posterior cerebellum has a greater number of significantly altered connections than the anterior cerebellum in the PTSD group. Mann-Whitney U tests confirmed that posterior cerebellar subregions exhibited a greater number of significant functional connections associated with PTSD severity (M = 13.93) than anterior subregions (M = 1.67; ***U*** = 76.5, *p* = 0.003). To test our hypothesis regarding the CEN, SN, and DMN, we ran the Mann-Whitney U tests for each network. The posterior cerebellar subregion connections with the DMN displayed a greater number of significant functional connections with PTSD than controls (***U*** = 72.0, *p* = 0.007), but no significant differences were observed with the CEN (***U*** = 55.5, *p* = 0.131) or SN (***U*** = 57.5, *p* = 0.079). Supplementary analyses were conducted to examine the potential influence of comorbid MDD diagnosis as a fixed covariate in PTSD diagnois and PTSD severity. There were no functional connectivity differences.

**Fig. 1:**
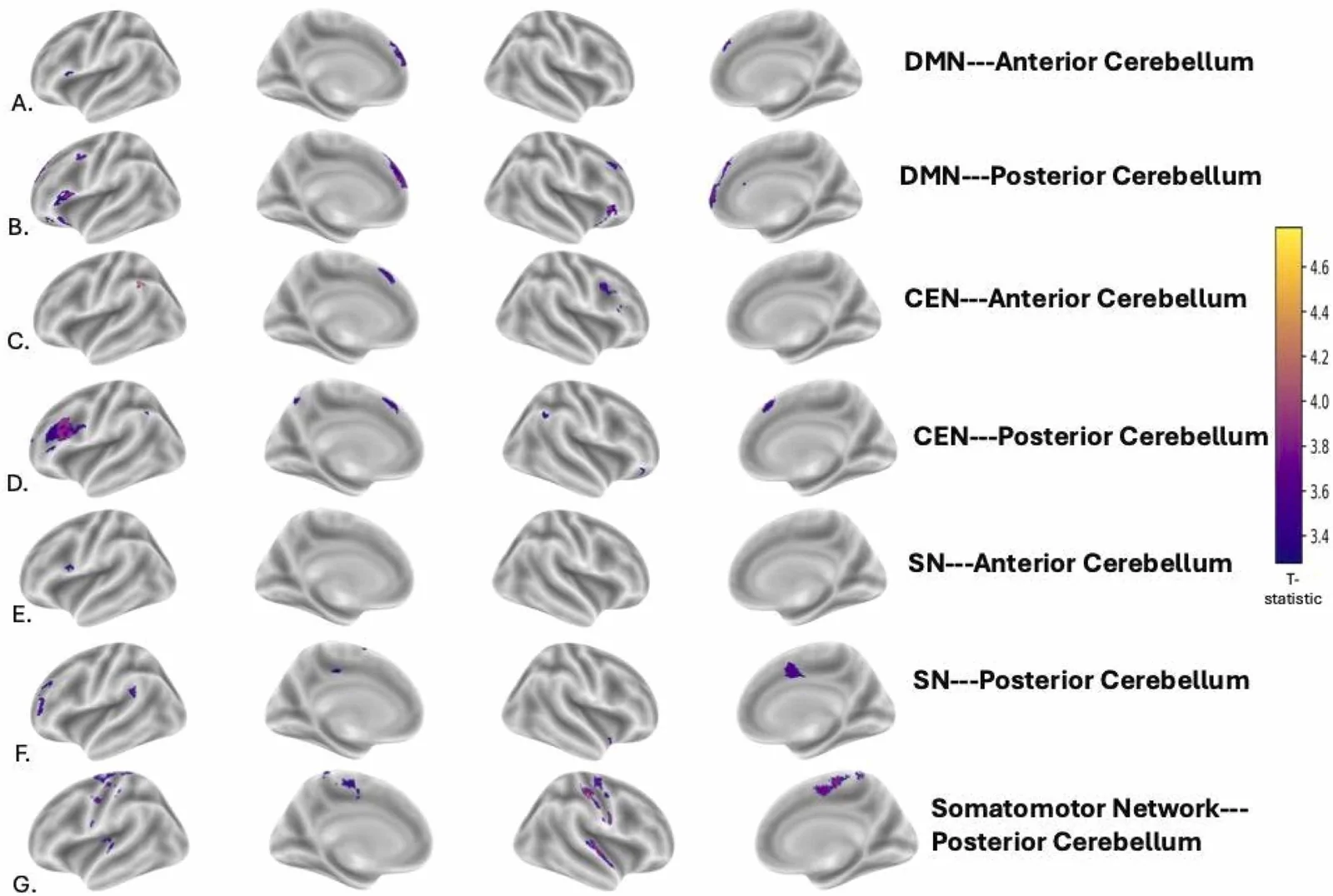
.

Fig. 1 shows greater resting-state functional connectivity of anterior and posterior cerebellum with large-scale cortical networks (DMN, CEN, salience, and somatomotor) in individuals with PTSD compared to controls.

## Discussion

The overall goal of our study was to examine resting-state functional connectivity of the anterior and posterior cerebellum with canonical networks in PTSD. We investigated this question using linear mixed-effects models to test whether PTSD alters the functional connectivity of major divisions of the cerebellum (anterior and posterior) with canonical resting-state networks. The findings suggest that PTSD diagnosis was primarily associated with posterior cerebellar connectivity with the default mode (DMN), whereas PTSD severity was strongly associated with posterior cerebellar connectivity with the central executive (CEN), salience (SN), somatomotor (SOM), and limbic networks relative to the anterior cerebellum. Our findings provided partial support for our hypotheses. Although there was an association of PTSD diagnosis with connectivity between the DMN and posterior cerebellum, diagnostic group differences were not found for posterior cerebellar connectivity with the CEN and SN. By contrast to PTSD diagnosis, our findings showed that PTSD symptom severity attested to broader posterior cerebellar connectivity disruptions with multiple networks, including DMN, SN, and CEN.

The DMN is responsible for recollection of personal experiences and daydreaming, CEN for cognitive processing and directed attention, and SOM for sensory and motor formation to execute purposeful movements [48], [49], [50], [51]. Altered connectivity between these networks and the cerebellum may be associated with cognitive, emotional and physiological dysfunction in PTSD. Previous work has suggested that PTSD is associated with altered cerebellar interactions with large-scale cortical networks. For example, in a smaller sample, Vuper and colleagues (2021) found that individuals with PTSD exhibited reduced CEN-cerebellum connectivity relative to trauma-exposed controls prior to treatment, which was normalized after participating in Cognitive Processing Therapy (CPT). In addition, connectivity between the DMN and SN as well as between the DMN and cerebellum was associated with PTSD symptoms. Their work highlights whole cerebellar involvement in PTSD and network connectivity in relation to treatment response. Similarly, Holmes and colleagues (2018) reported reduced cerebellar volume and weaker connectivity between the CEN nodes and cerebellar and DMN regions which was linked to greater PTSD severity. Other published work using rsFC revealed that the PTSD dissociative subtype is related to decreased functional connectivity between the anterior cerebellum and multiple sensorimotor regions compared to non-dissociative PTSD and trauma-naive controls [14]. In contrast, our current findings showed network disruptions between large-scale networks and the cerebellum which may suggest that cerebellar-network disruptions vary in direction and magnitude across networks. Notably, we observed these effects differentially impacted posterior cerebellar connectivity in a much larger sample spanning multiple cohorts with heterogeneous trauma types and diverse sociodemographic backgrounds. Together, our findings indicate that PTSD is associated with a greater number of altered posterior cerebellum connections with large-scale networks. Although previous studies have identified altered-cerebellar structure and connectivity in PTSD, it’s unclear whether these alterations represent pre-existing vulnerability factors or if they were associated with trauma exposure and PTSD diagnosis.

PTSD has been conceptualized as a disorder involving the amygdala, hippocampus, and prefrontal cortical regions in emotional regulation, memory and fear processing. Rather than suggesting that the cerebellum is an independent mechanism, our findings support the view that it functions as an integrated component of these broader large-scale networks. Given that there are reciprocal connections with cortical regions, the cerebellum may contribute to circuitry that is fundamental in emotional regulation, cognitive control, self-referential processing and salience detection. Our study extends the existing neurobiological models of PTSD highlighting the cerebellum as another contributor to network-level dysfunction. Given the high comorbidity between PTSD and MDD, it’s not surprising that there’s were no functional connectivity differences after adding an MDD covariate. The considerable clinical overlap between PTSD and MDD means that two regressors that share considerable variance impedes the ability to discover PTSD-related associations after accounting for MDD diagnosis. Cerebellar alterations represent a broader pattern of PTSD related structural dysregulation with evidence of lower total volume and cerebellar subregional volumes in PTSD as reported in Huggins et al. [11]. Nevertheless, the dense connectivity of the posterior cerebellum with prefrontal cortical regions in PTSD propose that cerebellar measures could provide additional information about network dysfunction. Whether these measures provide predictions beyond markers like the hippocampal or amygdala volume can be investigated through direct comparison in future studies.

An improved understanding of cortico-cerebellar connectivity in PTSD may serve to elucidate mechanisms that alleviate chronic symptoms of PTSD. This may also facilitate developing targeted interventions with improved efficacy. For instance, cognitive processing therapy (CPT) produces symptom improvements in PTSD and has been associated with decreased CEN-cerebellum connectivity and decreased DMN connectivity, while decreased rumination was associated with altered SN connectivity [33]. To investigate treatment targets, neuromodulation with transcranial magnetic stimulation (TMS) can be used to stimulate the site directly as well as sites that are anatomically or functionally connected [52]. Paired associative stimulation may induce plasticity when applied with a behavioral task, selectively enhancing cortico-cerebellar synaptic efficiency [53]. A paradigm to strengthen these connections may involve threat processing, particularly stimuli that evoke a fight-or-flight response [54]. Stimulation of cerebellar Crus I–II can reduce contralateral motor cortex activity during emotional face viewing, while motor cerebellar inhibition increases motor evoked potentials [55]. TMS of the lateral posterior cerebellum appears specific to emotional stimuli and may influence frontal and temporal regions involved in emotion discrimination [56], [57]. Neuromodulation of the posterior cerebellum has been shown to engage limbic, frontoparietal, and default mode networks, thereby supporting TMS treatment development for PTSD by informing identification and remediation of altered connectivity [58], [59], [60], [61]).

## Limitations

While our study promotes the understanding of cortico-cerebellar connectivity in PTSD, it is important to acknowledge its limitations. Although PTSD diagnosis and severity are associated with altered cortico-cerebellar connectivity, the causality of the relationships cannot be determined. There is uncertainty in whether the connectivity alterations contribute to constant symptom expression, or if PTSD causes a direct disruption within the cerebellar networks. There was limited availability regarding psychotropic medication use, alcohol consumption and substance use across participants. There was also an unequal distribution of comorbid Major Depressive Disorder (MDD) across sites. Additionally, PTSD classification was based on different assessment methods across the sites. Some participants were categorized based on validated self-report measures (PCL-5, PSS4) rather than clinician-administered diagnostic assessments which could have introduced variability in PTSD classification across sites. Although previous structural findings influenced the present study, cerebellar volumes were not examined as potential predictors of functional connectivity differences. Future studies may benefit by incorporating both structural and functional measures, which could determine if individual differences in cerebellar structure are associated with alterations in corticocerebellar connectivity. Our study was cross-sectional and potentially this limitation along with others, highlights the importance of longitudinal studies with consistent clinical characterization to ensure the clinical relevance of cortico-cerebellar alterations in PTSD.

## Conclusion

PTSD was associated with disruptions in anterior and posterior cerebellar connectivity with various networks involved in executive control, self-referential processing, salience detection, and sensorimotor integration. The increased connectivity strength between the posterior cerebellum and the default mode, central executive and salience networks in PTSD may be related to core PTSD symptoms including hyperarousal, intrusive memories, and emotional dysregulation. Mapping the relationship between cerebellar divisions and large-scale networks provide a framework for generating models of therapeutic targets and circuit level dysfunctions.

## Ethics declaration

Written informed consent to take part in the study and to publish the article has been obtained from all participants or their legal representatives. The privacy rights of participants have been observed.

This study was performed in compliance with relevant laws, regulatory frameworks and guidelines where the research took place. The Duke Health System IRB granted an exemption. The authors provided the following explanation: Exempt because it was a secondary data analysis

## Case Reports

This research follows the CARE guidelines and the CARE checklist.

## Declaration of Competing Interest

The authors declare that they have no known competing financial interests or personal relationships that could have appeared to influence the work reported in this paper.
