# Supplementary material for: Differential resting-state functional connectivity of anterior and posterior cerebellar divisions with canonical networks in PTSD
Source: J Mood Anxiety Disord. 2026 Oct 20;16:100209. doi: 10.1016/j.xjmad.2026.100209 (PMC13718838; doi:10.1016/j.xjmad.2026.100209)
Supplement: Supplementary file 1 [file mmc1.docx]

**SUPPLEMENTARY INFORMATION**

**Differential Resting-State Functional Connectivity of Anterior and Posterior Cerebellar Divisions with Canonical Networks in PTSD**

Tunstall et al.

**Table S1: Site-Specific Demographics**

**Table S2: Inclusion & Exclusion Criteria**

| Site | PI(s) | Location | Inclusion Criteria | Exclusion Criteria | Trauma Exposure |
| --- | --- | --- | --- | --- | --- |
| AURORA | Stevens, Ressler, McLean, Koenen, Kessler | Belmont, MA, USA; Detroit, MI, USA; Philadelphia, PA, USA; St. Louis, MO, USA; Atlanta, GA, USA | Emergency department patients enrolled within 72 hours of trauma (with MRI scans occurring at 6 months post-trauma), ages 18–75, able to speak and read English, oriented to time and place, physically able to use a smartphone, and possessed a smartphone for more than 1 year. Eligible types of trauma exposures: Motor vehicle collision, physical assault, sexual assault, fall greater than 10 feet, or mass casualty incidents; additional events involving actual or threatened death or serious injury. | Intracranial injury, long bone fracture, solid organ injury > grade 1, significant hemorrhage, required a chest tube or general anesthesia, or were likely to be admitted for >72 hours. Visual or auditory impairments that would preclude completion of web-based neurocognitive evaluations, prisoners, pregnancy, ongoing domestic violence, presenting to the ED after an intentional self-inflicted injury or suicide attempt. Additional MRI-related exclusion criteria: metal implant or foreign body; tattoo or tattooed makeup; sickle cell anemia, neurological disorder, or latex allergy. |  |
| Beijing | Li | Beijing, China | Individuals 18-65 years of age who personally experienced Wenchuan earthquake in 2008 and are right-handed | Intellectual disability; major psychosis (e.g., schizophrenia and organic mental disorders); drug or alcohol abuse; history of head trauma or surgery; metallic embedded object in body; claustrophobia; exposure to other trauma events from time of the disaster to the time of the study. | Inclusion criteria |
| Duke | Morey | Durham, NC, USA | Veterans 18-65 years of age, fluent in English, free of implanted metal objects or metal shards in eyes | Axis I psychiatric conditions other than PTSD or MDD; current substance abuse or lifetime substance dependence (other than nicotine); high risk for suicide, claustrophobial; neurological disorders; learning disability or developmental delay; major medical conditions | Military |
| Emory | Stevens,  Fani | Atlanta, GA, USA | Veterans 18-65 years of age, fluent in English, free of implanted metal objects or metal shards in eyes | Axis I psychiatric conditions other than PTSD or MDD; current substance abuse or lifetime substance dependence (other than nicotine); high risk for suicide, claustrophobial; neurological disorders; learning disability or developmental delay; major medical conditions | Inclusion criteria |
| Emory-ED | Stevens, Ressler | Atlanta, GA, USA | Emergency department patients enrolled within 24 hours of a traumatic event (with MRI scans occurring at 6 months post-trauma), 18 to 65 years of age, English-speaking. Qualifying traumas were defined by the DSM-IV-TR Criterion A for PTSD. | Current psychotic symptoms or bipolar disorder; current substance dependence; history of head trauma; psychoactive medication usage; current illegal drug use (verified with urine drug screen within 24 hours of scan) | Inclusion criteria |
| Groningen | Daniels | Groningen, Netherlands | Civilian women 20-60 years of age with a current PTSD diagnosis, sufficient proficiency in German, MRI compatible | Neurologic disorders; history of substance abuse or dependence (past 6 months); history of head injury; cerebral incidental findings verified by a neuroradiologist after the MR scan; usage of benzodiazepines, tricyclic antidepressants, or anticonvulsants; primary borderline personality disorder; other current diagnosis of Axis I disorder | PTSD only |
| South Dakota | Baugh,  Fercho | Vermillion, SD, USA | OIF/ OEF/OND veterans | Current or previous seizure history; current crisis-related issues such as serious self-injurious behavior, psychosis, or substance dependence (excluding alcohol dependence); report of traumatic brain injury using the Traumatic Brain Injury Checklist (Hoge et al., 2008); contraindications to fMRI (metal objects in body, claustrophobia) | Military |
| Wisconsin-Grupe | Grupe | Madison, WI, USA | Adults 18-50 with exposure to 1+ life-threatening war zone trauma events; capable of giving informed consent and fluent in English; clear evidence of war zone trauma exposure in Iraq or Afghanistan since 2001 (e.g., Combat Action Ribbon [Marines], Combat Infantry Badge [Army]); stable pharmacological or psychotherapeutic treatment for at least 8 weeks prior to beginning of study | Weight >352 pounds or over; pregnancy or current breastfeeding; Metallic implants such as prostheses or aneurysm clip, or electronic implants such as cardiac pacemakers; Neurological or serious medical condition; History of seizures or seizure disorder; Moderate or severe traumatic brain injury; Current active substance dependence or dependence within 3 months (other than nicotine); bipolar disorder, schizophrenia, schizoaffective disorder, psychotic disorder NOS, delirium, or any DSM-IV cognitive disorder; Severe psychiatric instability or severe situational life crises, (e.g., suicidality, homicidality); extensive experience in yoga or meditation; Current use of benzodiazepines and beta-blockers | Inclusion criteria |

***Table 3: Scanner Information by site***

| Site | Scanner | Field  strength | Volumes | # head  coil  channels | Sequence | Voxel size (mm) | FOV (mm) | Orientation | Repetition time (TR) | Echo time (TE) | Flip angle |
| --- | --- | --- | --- | --- | --- | --- | --- | --- | --- | --- | --- |
| AURORA | Siemens TIM Trio | 3T | 230 | 12 | EPI | 3x2.72x2.72 | 212x212 | Interleaved | 2360 | 30 | 70 |
|  | Siemens TIM Trio | 3T | 230 | 12 | EPI | 3x3x3 | 212x212 | Interleaved | 2360 | 30 | 70 |
|  | Siemens MAGNETOM Prisma | 3T | 230 | 20 | EPI | 3x2.72x2.72 | 212x212 | Interleaved | 2360 | 29 | 70 |
|  | Siemens Verio | 3T | 230 | 12 | EPI | 3x2.72x2.72 | 212x212 | Interleaved | 2360 | 30 | 70 |
|  | Siemens MAGNETOM Prisma | 3T | 230 | 12 | EPI | 3x3x2.5 | 210x210 | Interleaved | 2360 | 29 | 90 |
| Beijing | Philips  Achieva | 1.5T | 150 | NA | NA | 3.44x3.44x4 | 220x220 | Axial | 3000 | 30 | 90 |
| Duke | GE MR750 | 3T | 176 | 8 | SENSE Spiral | 3.75x3.75x4 | 240x240 | Axial oblique | 2000 | 30 | 60 |
|  | GE MR750 | 3T | 196 | 8 | SENSE Spiral | 4x4x3.8 | 256x256 | Axial | 2000 | 27 | 60 |
|  | Philips Ingenia | 3T | 176 | NA | Gradient Recalled, segmented k-space | 1.8x1.8x4 | 230x230 | Axial oblique | 2000 | NA | 90 |
|  | GE MR750 | 3T | 170 | 8 | SENSE Spiral | 3.75x3.75x4 | 240x240 | Axial oblique | 2000 | 30 | 60 |
|  | GE Signa Excite | 3T | 170 | 8 | SENSE Spiral | 3.75x3.75x4 | 240x240 | Axial oblique | 2000 | 30 | 60 |
| Emory | Siemens Trio | 3T | 150 | 12 | EPI | 3.4x3.4x4 | 220X220 | Interleaved | 2950 | 30 / 67 | 90 |
| Emory-ED | Siemens TrioTim | 3T | 150 | 12 | EPI | 3x3x2.5 | 220x220 | Axial | 2360 | 30 | 70 |
|  | Siemens TrioTim | 3T | NA | NA | Z-SAGA | 3.4x3.4x4 | 220x220 | Axial | 2950 | 30 | 90 |
|  | Siemens TrioTim | 3T | NA | NA | Z-SAGA | 3.4x3.4x4 | 220x220 | Axial | 2950 | 30 | 90 |
| Groningen | Siemens MAGNETOM TrioTim syngo MR B17 | 3T | 132 | 12 | EPI | 3x3x3 | 192x192 | Transversal | 2250 | 25 | 80 |
| South Dakota | Siemens Skyra | 3T | 387 | 20 | NA | 3.40x3.40x4 | 240x240 | NA | 2000 | 30 | NA |
| Wisconsin-Grupe | GE X750 Discovery | 3T | 206 | 8 | EPI | 1.719x1.719x4 | 220x220 | Sagittal | 2000 | 20 | 60 |

***Table S4: Details for PTSD assessments***

| Item | Abbreviation | # Questions | Scale | Range | Adjusted Max |
| --- | --- | --- | --- | --- | --- |
| Modified PTSD Symptom Scale (MPSS-SR) | MPSS | 17 | 0–3 | 0–51 | 51 |
| PTSD Symptom Scale – Self Report (PSS-SR) | PSS | 17 | 0–3 | 0–51 | 51 |
| PTSD Checklist for DSM-5 (PCL-5) | PCL-5 | 20 | 0–4 | 0–80 | 80 |
| Clinician-Administered PTSD Scale for DSM-5 (CAPS-5) | CAPS-5 | 20 | 0–4 | 0–80 | 80 |
| Clinician-Administered PTSD Scale for DSM-4 (CAPS-4) | CAPS-4 | 17 | 0–8 | 0–136 | 136 |

***Table S5: Race & Ethnicity data by site***

| **Site** | **Race** | | | | | | | | **Ethnicity** | | |
| --- | --- | --- | --- | --- | --- | --- | --- | --- | --- | --- | --- |
|  | **White** | **Asian** | **Black** | **Other** | **Multi-racial** | **Native-**  **American** | **Pacific Islander** | **Not Reported** | **H** | **NH** | **Not Reported** |
| AURORA | 46 | 6 | 62 | 0 | 0 | 0 | 0 | 19 | 19 | 115 | 0 |
| Beijing | 0 | 77 | 0 | 0 | 0 | 0 | 0 | 0 | 0 | 77 | 0 |
| Duke | 61 | 0 | 53 | 7 | 0 | 0 | 0 | 11 | 4 | 89 | 39 |
| Emory | 0 | 0 | 58 | 1 | 0 | 0 | 0 | 0 | 0 | 59 | 0 |
| Emory-ED | 8 | 0 | 20 | 0 | 1 | 0 | 0 | 0 | 0 | 29 | 0 |
| Groningen | 35 | 0 | 0 | 0 | 0 | 0 | 0 | 0 | 1 | 34 | 0 |
| South Dakota | 16 | 1 | 0 | 1 | 1 | 0 | 0 | 0 | 1 | 0 | 19 |
| Wisconsin-Madison –Grupe  ***Table S6: PTSD Severity Significant Connections*** | 52 | 0 | 1 | 0 | 0 | 0 | 1 | 1 | 2 | 52 | 1 |

| **#** | **Node 1** | **Node 2** | **Statistic** | ***p*_FDR_** |
| --- | --- | --- | --- | --- |
| 1 | 17Networks_LH_ContA_PFCl_2 | Left_VIIIB_Post | 4.775 | 0.020 |
| 2 | 17Networks_LH_DefaultB_PFCv_5 | Right_VIIB_Post | 4.594 | 0.020 |
| 3 | 17Networks_RH_DefaultB_PFCd_1 | Left_X | 4.506 | 0.020 |
| 4 | 17Networks_RH_SomMotA_4 | Left_Crus_II_Post | 4.486 | 0.020 |
| 5 | 17Networks_RH_SomMotB_Cent_2 | Left_X | 4.424 | 0.020 |
| 6 | 17Networks_LH_DefaultB_PFCv_5 | Left_VIIIB_Post | 4.399 | 0.020 |
| 7 | 17Networks_LH_SomMotB_Cent_3 | Left_X | 4.360 | 0.020 |
| 8 | 17Networks_LH_DefaultB_PFCv_5 | Right_VIIIB_Post | 4.327 | 0.020 |
| 9 | 17Networks_RH_SomMotB_Aud_1 | Left_Crus_II_Post | 4.296 | 0.020 |
| 10 | 17Networks_LH_ContB_IPL_2 | Left_V_Ant | 4.284 | 0.020 |
| 11 | 17Networks_LH_ContA_PFClv_1 | Corpus_Medullare | 4.277 | 0.020 |
| 12 | 17Networks_RH_DefaultB_PFCd_4 | Right_X | 4.267 | 0.020 |
| 13 | 17Networks_RH_DefaultA_PFCm_4 | Left_VIIIB_Post | 4.256 | 0.020 |
| 14 | 17Networks_LH_DefaultB_PFCd_3 | Left_VIIIB_Post | 4.252 | 0.020 |
| 15 | 17Networks_LH_DefaultB_PFCv_5 | Corpus_Medullare | 4.234 | 0.020 |
| 16 | 17Networks_RH_SomMotA_13 | Left_Crus_II_Post | 4.210 | 0.020 |
| 17 | 17Networks_RH_SomMotA_11 | Right_Crus_II_Post | 4.210 | 0.020 |
| 18 | 17Networks_RH_DefaultB_PFCd_3 | Left_X | 4.201 | 0.020 |
| 19 | 17Networks_LH_DefaultB_PFCv_5 | Left_VIIIA_Post | 4.191 | 0.020 |
| 20 | 17Networks_RH_DefaultB_PFCd_1 | Right_X | 4.186 | 0.020 |
| 21 | 17Networks_RH_SomMotA_7 | Left_Crus_II_Post | 4.184 | 0.020 |
| 22 | 17Networks_RH_DefaultB_PFCv_2 | Left_Crus_II_Post | 4.179 | 0.020 |
| 23 | 17Networks_RH_DorsAttnB_PostC_4 | Left_Crus_II_Post | 4.176 | 0.020 |
| 24 | 17Networks_RH_SomMotA_11 | Left_X | 4.174 | 0.020 |
| 25 | 17Networks_RH_SomMotA_11 | Left_Crus_II_Post | 4.145 | 0.021 |
| 26 | 17Networks_LH_DefaultB_PFCv_1 | Left_VIIB_Post | 4.102 | 0.024 |
| 27 | 17Networks_RH_SomMotA_11 | Right_VIIB_Post | 4.090 | 0.024 |
| 28 | 17Networks_RH_SomMotB_Aud_2 | Left_Crus_II_Post | 4.089 | 0.024 |
| 29 | 17Networks_LH_DorsAttnA_TempOcc_4 | Left_VIIB_Post | 4.068 | 0.025 |
| 30 | 17Networks_LH_DefaultB_PFCv_5 | Right_VI_Post | 4.057 | 0.025 |
| 31 | 17Networks_RH_DefaultB_PFCd_3 | Right_VIIB_Post | 4.050 | 0.025 |
| 32 | 17Networks_LH_TempPar_5 | Right_VIIIB_Post | 4.029 | 0.026 |
| 33 | 17Networks_LH_DefaultB_PFCd_1 | Right_VIIB_Post | 4.022 | 0.026 |
| 34 | 17Networks_LH_DefaultB_PFCd_1 | Right_VIIIB_Post | 4.018 | 0.026 |
| 35 | 17Networks_RH_DefaultB_PFCd_3 | Right_X | 4.017 | 0.026 |
| 36 | 17Networks_RH_DorsAttnB_PostC_1 | Left_Crus_II_Post | 4.002 | 0.026 |
| 37 | 17Networks_LH_TempPar_5 | Left_VIIIB_Post | 3.998 | 0.026 |
| 38 | 17Networks_RH_SomMotA_13 | Right_Crus_II_Post | 3.986 | 0.026 |
| 39 | 17Networks_RH_SomMotB_Cent_2 | Left_Crus_II_Post | 3.985 | 0.026 |
| 40 | 17Networks_RH_SomMotB_Aud_2 | Right_Crus_II_Post | 3.981 | 0.026 |
| 41 | 17Networks_LH_DefaultB_PFCv_5 | Left_VI_Post | 3.975 | 0.027 |
| 42 | 17Networks_LH_DefaultB_PFCd_1 | Left_VIIIB_Post | 3.965 | 0.028 |
| 43 | 17Networks_LH_DefaultB_PFCd_1 | Right_X | 3.941 | 0.029 |
| 44 | 17Networks_LH_SomMotA_13 | Left_Crus_II_Post | 3.936 | 0.029 |
| 45 | 17Networks_LH_SalVentAttnA_FrOper_2 | Corpus_Medullare | 3.927 | 0.029 |
| 46 | 17Networks_RH_ContB_PFCmp_1 | Right_VIIB_Post | 3.914 | 0.029 |
| 47 | 17Networks_LH_DefaultB_PFCd_4 | Left_X | 3.904 | 0.029 |
| 48 | 17Networks_RH_DefaultB_PFCd_3 | Vermis_VI | 3.903 | 0.029 |
| 49 | 17Networks_LH_DefaultB_PFCd_4 | Left_VIIIB_Post | 3.901 | 0.029 |
| 50 | 17Networks_RH_DefaultB_PFCd_2 | Right_VIIB_Post | 3.895 | 0.029 |
| 51 | 17Networks_RH_DefaultB_PFCd_3 | Left_VIIIB_Post | 3.894 | 0.029 |
| 52 | 17Networks_RH_DefaultA_PFCm_4 | Right_X | 3.886 | 0.029 |
| 53 | 17Networks_RH_SomMotA_6 | Left_Crus_II_Post | 3.881 | 0.029 |
| 54 | 17Networks_LH_DefaultB_PFCv_5 | Left_X | 3.878 | 0.029 |
| 55 | 17Networks_LH_SomMotA_5 | Left_Crus_II_Post | 3.875 | 0.029 |
| 56 | 17Networks_LH_DefaultB_PFCd_1 | Vermis_X | 3.862 | 0.029 |
| 57 | 17Networks_RH_DefaultA_PFCm_4 | Right_VIIB_Post | 3.861 | 0.029 |
| 58 | 17Networks_LH_DefaultB_PFCv_5 | Left_VIIB_Post | 3.852 | 0.030 |
| 59 | 17Networks_LH_DefaultA_PFCd_2 | Left_VIIIB_Post | 3.846 | 0.030 |
| 60 | 17Networks_RH_ContB_PFClv_1 | Left_Crus_II_Post | 3.836 | 0.031 |
| 61 | 17Networks_LH_DefaultB_PFCd_1 | Left_X | 3.802 | 0.035 |
| 62 | 17Networks_LH_SomMotB_Cent_5 | Left_X | 3.799 | 0.035 |
| 63 | 17Networks_RH_DefaultB_PFCd_3 | Right_V_Ant | 3.796 | 0.035 |
| 64 | 17Networks_LH_TempPar_5 | Left_X | 3.790 | 0.036 |
| 65 | 17Networks_RH_DefaultA_PFCm_4 | Left_VIIIA_Post | 3.778 | 0.036 |
| 66 | Right-Amygdala | Left_IX_Post | 3.764 | 0.036 |
| 67 | 17Networks_RH_SomMotA_5 | Left_Crus_II_Post | 3.763 | 0.036 |
| 68 | 17Networks_RH_TempPar_1 | Corpus_Medullare | 3.763 | 0.036 |
| 69 | 17Networks_LH_SomMotA_18 | Left_VI_Post | 3.753 | 0.036 |
| 70 | 17Networks_RH_LimbicB_OFC_4 | Vermis_VII | 3.744 | 0.036 |
| 71 | 17Networks_RH_ContA_IPS_1 | Vermis_VIII | 3.743 | 0.036 |
| 72 | 17Networks_RH_DefaultB_Temp_2 | Corpus_Medullare | 3.742 | 0.036 |
| 73 | 17Networks_LH_SomMotB_Cent_4 | Left_Crus_II_Post | 3.737 | 0.036 |
| 74 | 17Networks_LH_SomMotA_9 | Left_Crus_II_Post | 3.736 | 0.036 |
| 75 | 17Networks_RH_DefaultB_PFCv_1 | Left_VIIB_Post | 3.736 | 0.036 |
| 76 | 17Networks_RH_DorsAttnB_PostC_2 | Left_Crus_II_Post | 3.732 | 0.037 |
| 77 | 17Networks_RH_SomMotB_Aud_2 | Right_X | 3.722 | 0.037 |
| 78 | 17Networks_LH_ContA_PFCl_2 | Left_VIIIA_Post | 3.714 | 0.038 |
| 79 | 17Networks_RH_DefaultB_PFCd_2 | Left_VIIIB_Post | 3.708 | 0.038 |
| 80 | 17Networks_RH_DefaultB_PFCd_4 | Left_X | 3.705 | 0.038 |
| 81 | 17Networks_LH_ContA_PFClv_1 | Left_VI_Post | 3.698 | 0.039 |
| 82 | 17Networks_LH_SomMotA_7 | Left_Crus_II_Post | 3.693 | 0.039 |
| 83 | Left_V_Ant | Left_VIIB_Post | 3.689 | 0.039 |
| 84 | 17Networks_RH_DorsAttnB_PostC_3 | Left_Crus_II_Post | 3.680 | 0.040 |
| 85 | 17Networks_RH_DefaultB_PFCd_3 | Left_VIIB_Post | 3.677 | 0.040 |
| 86 | 17Networks_LH_SomMotA_13 | Right_Crus_II_Post | 3.670 | 0.040 |
| 87 | 17Networks_RH_SomMotA_14 | Left_Crus_II_Post | 3.669 | 0.040 |
| 88 | 17Networks_LH_DefaultB_PFCd_1 | Vermis_IX | 3.668 | 0.040 |
| 89 | Right_Crus_II_Post | Vermis_VIII | 3.659 | 0.040 |
| 90 | 17Networks_RH_SomMotA_14 | Right_Crus_II_Post | 3.649 | 0.040 |
| 91 | 17Networks_LH_SalVentAttnA_ParOper_2 | Left_VIIB_Post | 3.644 | 0.040 |
| 92 | 17Networks_RH_LimbicB_OFC_4 | Left_VI_Post | 3.642 | 0.040 |
| 93 | 17Networks_RH_DefaultB_Temp_1 | Vermis_VII | 3.640 | 0.040 |
| 94 | 17Networks_RH_DefaultB_PFCd_3 | Right_VI_Post | 3.639 | 0.040 |
| 95 | 17Networks_RH_DefaultA_PFCm_4 | Left_VIIB_Post | 3.638 | 0.040 |
| 96 | 17Networks_RH_SomMotA_2 | Left_Crus_II_Post | 3.633 | 0.040 |
| 97 | 17Networks_RH_DorsAttnB_PostC_5 | Left_Crus_II_Post | 3.633 | 0.040 |
| 98 | 17Networks_LH_SalVentAttnB_PFCl_1 | Right_VIIIB_Post | 3.630 | 0.040 |
| 99 | 17Networks_LH_DefaultB_PFCl_2 | Left_VIIIB_Post | 3.627 | 0.040 |
| 100 | 17Networks_RH_DefaultB_PFCv_2 | Right_VIIB_Post | 3.624 | 0.040 |
| 101 | 17Networks_RH_DorsAttnA_SPL_6 | Right_Crus_II_Post | 3.624 | 0.040 |
| 102 | 17Networks_LH_SomMotA_18 | Left_Crus_II_Post | 3.624 | 0.040 |
| 103 | 17Networks_RH_DefaultB_AntTemp_1 | Vermis_VIII | 3.623 | 0.040 |
| 104 | 17Networks_LH_SalVentAttnA_ParOper_2 | Left_VIIIA_Post | 3.619 | 0.040 |
| 105 | 17Networks_RH_SomMotB_Aud_1 | Left_VIIB_Post | 3.617 | 0.040 |
| 106 | 17Networks_LH_DefaultB_PFCd_1 | Right_I-III_Ant | 3.610 | 0.040 |
| 107 | 17Networks_RH_ContB_IPL_1 | Vermis_VI | 3.610 | 0.040 |
| 108 | 17Networks_LH_SalVentAttnB_PFCl_2 | Left_VIIIB_Post | 3.608 | 0.040 |
| 109 | Left_V_Ant | Left_VIIIB_Post | 3.607 | 0.040 |
| 110 | 17Networks_LH_DefaultB_PFCv_5 | Right_Crus_II_Post | 3.605 | 0.040 |
| 111 | 17Networks_RH_SalVentAttnA_Ins_1 | Left_VIIB_Post | 3.605 | 0.040 |
| 112 | 17Networks_RH_ContA_PFCl_5 | Vermis_VI | 3.600 | 0.040 |
| 113 | 17Networks_LH_TempPar_4 | Vermis_IX | 3.598 | 0.040 |
| 114 | 17Networks_RH_ContA_PFCl_2 | Right_X | 3.597 | 0.040 |
| 115 | 17Networks_RH_ContA_PFCl_5 | Right_V_Ant | 3.591 | 0.040 |
| 116 | 17Networks_RH_SalVentAttnA_FrMed_1 | Left_Crus_II_Post | 3.588 | 0.040 |
| 117 | 17Networks_RH_DefaultA_PFCm_4 | Right_VIIIA_Post | 3.588 | 0.040 |
| 118 | 17Networks_LH_ContA_PFCl_1 | Left_VIIIB_Post | 3.587 | 0.040 |
| 119 | 17Networks_LH_SomMotA_12 | Left_Crus_II_Post | 3.580 | 0.040 |
| 120 | 17Networks_LH_DorsAttnA_TempOcc_4 | Left_VIIIA_Post | 3.578 | 0.040 |
| 121 | 17Networks_LH_ContB_PFCmp_1 | Right_I-III_Ant | 3.574 | 0.040 |
| 122 | 17Networks_RH_TempPar_2 | Right_Crus_II_Post | 3.572 | 0.040 |
| 123 | 17Networks_LH_ContB_PFCmp_1 | Right_VIIB_Post | 3.572 | 0.040 |
| 124 | 17Networks_RH_ContB_PFClv_3 | Vermis_VII | 3.571 | 0.040 |
| 125 | 17Networks_LH_SomMotB_Cent_5 | Left_VIIIB_Post | 3.567 | 0.040 |
| 126 | 17Networks_LH_DefaultB_PFCv_1 | Left_Crus_II_Post | 3.567 | 0.040 |
| 127 | 17Networks_LH_SomMotA_5 | Left_Crus_I_Post | 3.565 | 0.040 |
| 128 | 17Networks_LH_SalVentAttnB_PFCl_1 | Left_VIIIB_Post | 3.558 | 0.041 |
| 129 | 17Networks_RH_DefaultB_Temp_2 | Left_Crus_II_Post | 3.557 | 0.042 |
| 130 | 17Networks_RH_DefaultB_Temp_1 | Vermis_VIII | 3.555 | 0.042 |
| 131 | 17Networks_LH_TempPar_5 | Left_VIIB_Post | 3.551 | 0.041 |
| 132 | 17Networks_LH_SomMotA_16 | Right_Crus_II_Post | 3.550 | 0.042 |
| 133 | 17Networks_LH_DefaultB_PFCd_1 | Left_VIIB_Post | 3.550 | 0.041 |
| 134 | 17Networks_LH_SomMotA_12 | Left_Crus_I_Post | 3.545 | 0.042 |
| 135 | 17Networks_RH_TempPar_1 | Left_Crus_II_Post | 3.539 | 0.042 |
| 136 | 17Networks_RH_SomMotA_11 | Right_X | 3.539 | 0.042 |
| 137 | 17Networks_RH_SomMotA_12 | Left_Crus_II_Post | 3.536 | 0.042 |
| 138 | 17Networks_LH_DefaultB_PFCd_4 | Right_VIIB_Post | 3.534 | 0.042 |
| 139 | 17Networks_LH_DefaultB_PFCl_1 | Right_X | 3.528 | 0.042 |
| 140 | 17Networks_LH_SomMotA_17 | Left_Crus_II_Post | 3.524 | 0.042 |
| 141 | 17Networks_LH_SomMotA_13 | Right_VI_Post | 3.524 | 0.042 |
| 142 | 17Networks_LH_ContA_PFCl_1 | Left_VIIIA_Post | 3.524 | 0.042 |
| 143 | 17Networks_LH_SomMotB_Ins_1 | Left_Crus_II_Post | 3.524 | 0.042 |
| 144 | 17Networks_RH_DorsAttnB_PostC_3 | Right_Crus_II_Post | 3.522 | 0.042 |
| 145 | 17Networks_RH_SomMotB_Aud_2 | Right_VIIB_Post | 3.522 | 0.042 |
| 146 | 17Networks_LH_SomMotA_16 | Left_Crus_II_Post | 3.521 | 0.042 |
| 147 | 17Networks_RH_SomMotB_Aud_3 | Left_Crus_II_Post | 3.515 | 0.042 |
| 148 | 17Networks_RH_DorsAttnB_PostC_4 | Left_Crus_I_Post | 3.515 | 0.042 |
| 149 | 17Networks_RH_DefaultB_PFCd_3 | Left_VIIIA_Post | 3.513 | 0.042 |
| 150 | 17Networks_LH_ContB_IPL_1 | Vermis_VII | 3.509 | 0.042 |
| 151 | Left_V_Ant | Right_VIIB_Post | 3.509 | 0.042 |
| 152 | 17Networks_LH_SomMotA_4 | Left_Crus_II_Post | 3.507 | 0.042 |
| 153 | 17Networks_RH_DefaultB_PFCd_3 | Right_VIIIA_Post | 3.502 | 0.042 |
| 154 | 17Networks_RH_VisCent_ExStr_11 | Left_Crus_II_Post | 3.500 | 0.042 |
| 155 | 17Networks_RH_ContB_PFClv_1 | Vermis_VIII | 3.498 | 0.042 |
| 156 | 17Networks_LH_SomMotA_1 | Left_X | 3.491 | 0.042 |
| 157 | 17Networks_RH_DefaultB_PFCd_2 | Left_X | 3.491 | 0.042 |
| 158 | 17Networks_RH_SomMotA_8 | Left_Crus_II_Post | 3.489 | 0.042 |
| 159 | 17Networks_RH_DefaultA_PFCd_1 | Left_VIIIB_Post | 3.489 | 0.042 |
| 160 | Right_V_Ant | Vermis_VIII | 3.489 | 0.042 |
| 161 | 17Networks_LH_DefaultB_PFCv_5 | Right_X | 3.487 | 0.042 |
| 162 | 17Networks_LH_ContC_pCun_3 | Left_Crus_II_Post | 3.484 | 0.042 |
| 163 | 17Networks_RH_SomMotA_5 | Left_X | 3.482 | 0.042 |
| 164 | 17Networks_LH_SomMotA_14 | Left_Crus_II_Post | 3.480 | 0.042 |
| 165 | 17Networks_LH_DefaultB_PFCv_5 | Right_VIIIA_Post | 3.476 | 0.042 |
| 166 | 17Networks_LH_SomMotB_Aud_2 | Left_VIIB_Post | 3.475 | 0.042 |
| 167 | 17Networks_LH_SalVentAttnA_Ins_1 | Left_VIIB_Post | 3.475 | 0.042 |
| 168 | 17Networks_RH_DefaultB_PFCd_2 | Right_X | 3.471 | 0.044 |
| 169 | 17Networks_RH_SomMotA_11 | Left_VIIB_Post | 3.470 | 0.043 |
| 170 | 17Networks_RH_TempPar_1 | Vermis_VIII | 3.466 | 0.043 |
| 171 | 17Networks_LH_ContA_PFClv_1 | Left_VIIIB_Post | 3.465 | 0.043 |
| 172 | 17Networks_RH_ContB_PFCmp_1 | Left_VIIIA_Post | 3.462 | 0.043 |
| 173 | Left_V_Ant | Left_Crus_II_Post | 3.461 | 0.043 |
| 174 | 17Networks_RH_SomMotA_1 | Vermis_X | 3.458 | 0.043 |
| 175 | 17Networks_RH_DefaultB_PFCd_3 | Corpus_Medullare | 3.458 | 0.043 |
| 176 | 17Networks_RH_DefaultB_PFCd_3 | Right_IV_Ant | 3.457 | 0.043 |
| 177 | 17Networks_LH_DorsAttnA_SPL_5 | Left_VI_Post | 3.455 | 0.044 |
| 178 | 17Networks_LH_DefaultB_PFCd_4 | Left_VIIB_Post | 3.451 | 0.043 |
| 179 | 17Networks_RH_VisCent_ExStr_8 | Left_Crus_I_Post | 3.451 | 0.043 |
| 180 | 17Networks_LH_SomMotA_6 | Left_Crus_II_Post | 3.449 | 0.044 |
| 181 | 17Networks_RH_DefaultB_Temp_2 | Vermis_VII | 3.448 | 0.044 |
| 182 | 17Networks_LH_DefaultB_PFCv_5 | Right_V_Ant | 3.443 | 0.044 |
| 183 | 17Networks_RH_DefaultA_PFCm_4 | Left_X | 3.443 | 0.044 |
| 184 | 17Networks_LH_ContB_PFCmp_1 | Left_VIIIB_Post | 3.435 | 0.046 |
| 185 | 17Networks_LH_DefaultB_PFCv_5 | Left_Crus_I_Post | 3.428 | 0.045 |
| 186 | 17Networks_RH_DefaultB_PFCd_3 | Right_VIIIB_Post | 3.423 | 0.047 |
| 187 | 17Networks_LH_SomMotA_13 | Right_Crus_I_Post | 3.423 | 0.046 |
| 188 | 17Networks_RH_SomMotA_10 | Left_X | 3.422 | 0.046 |
| 189 | 17Networks_LH_SomMotB_Cent_5 | Right_VIIIB_Post | 3.421 | 0.046 |
| 190 | 17Networks_RH_LimbicB_OFC_6 | Left_Crus_I_Post | 3.420 | 0.047 |
| 191 | 17Networks_RH_ContA_PFCl_1 | Right_V_Ant | 3.419 | 0.046 |
| 192 | 17Networks_RH_DorsAttnB_TempOcc_1 | Vermis_VIII | 3.416 | 0.046 |
| 193 | 17Networks_LH_DorsAttnA_SPL_5 | Left_Crus_I_Post | 3.413 | 0.047 |
| 194 | 17Networks_LH_SalVentAttnB_PFCl_2 | Left_IX_Post | 3.412 | 0.046 |
| 195 | 17Networks_RH_SomMotA_16 | Vermis_IX | 3.409 | 0.047 |
| 196 | 17Networks_RH_SomMotA_8 | Vermis_X | 3.408 | 0.047 |
| 197 | 17Networks_LH_DefaultB_PFCv_5 | Vermis_VII | 3.407 | 0.047 |
| 198 | 17Networks_LH_ContA_PFClv_2 | Left_VIIIB_Post | 3.404 | 0.047 |
| 199 | 17Networks_RH_SomMotA_8 | Left_Crus_I_Post | 3.403 | 0.047 |
| 200 | 17Networks_RH_DefaultB_PFCd_3 | Right_I-III_Ant | 3.401 | 0.047 |
| 201 | 17Networks_RH_ContB_PFCmp_1 | Left_VI_Post | 3.395 | 0.047 |
| 202 | 17Networks_LH_SomMotB_Cent_1 | Left_Crus_II_Post | 3.395 | 0.047 |
| 203 | 17Networks_RH_SomMotA_1 | Left_Crus_II_Post | 3.395 | 0.047 |
| 204 | 17Networks_RH_SomMotA_6 | Left_VIIB_Post | 3.394 | 0.047 |
| 205 | 17Networks_RH_VisCent_ExStr_8 | Right_Crus_I_Post | 3.389 | 0.047 |
| 206 | 17Networks_LH_SalVentAttnA_FrOper_2 | Left_V_Ant | 3.389 | 0.047 |
| 207 | 17Networks_RH_TempPar_8 | Left_Crus_II_Post | 3.388 | 0.047 |
| 208 | 17Networks_RH_DefaultB_PFCd_2 | Left_IX_Post | 3.388 | 0.047 |
| 209 | 17Networks_LH_TempPar_5 | Left_VIIIA_Post | 3.382 | 0.047 |
| 210 | 17Networks_LH_LimbicB_OFC_2 | Left_Crus_I_Post | 3.381 | 0.048 |
| 211 | 17Networks_RH_DefaultA_PFCd_1 | Right_X | 3.377 | 0.048 |
| 212 | 17Networks_RH_SomMotA_11 | Right_VI_Post | 3.374 | 0.048 |
| 213 | 17Networks_RH_DefaultA_PFCm_2 | Left_Crus_I_Post | 3.373 | 0.048 |
| 214 | 17Networks_RH_SomMotA_3 | Left_Crus_II_Post | 3.369 | 0.048 |
| 215 | 17Networks_LH_SalVentAttnB_PFCl_2 | Left_Crus_II_Post | 3.368 | 0.048 |
| 216 | Right-Amygdala | Left_Crus_II_Post | 3.366 | 0.048 |
| 217 | 17Networks_LH_SomMotA_17 | Left_Crus_I_Post | 3.362 | 0.048 |
| 218 | 17Networks_RH_ContB_PFCmp_1 | Left_VIIB_Post | 3.360 | 0.048 |
| 219 | 17Networks_LH_SomMotA_18 | Right_VI_Post | 3.359 | 0.048 |
| 220 | 17Networks_RH_ContB_Temp_2 | Vermis_VIII | 3.358 | 0.048 |
| 221 | 17Networks_RH_SomMotB_Aud_1 | Right_Crus_II_Post | 3.358 | 0.048 |
| 222 | 17Networks_LH_DefaultB_PFCv_5 | Vermis_VIII | 3.356 | 0.048 |
| 223 | 17Networks_LH_SomMotA_1 | Left_Crus_II_Post | 3.355 | 0.048 |
| 224 | 17Networks_RH_DorsAttnA_SPL_7 | Left_Crus_II_Post | 3.352 | 0.048 |
| 225 | 17Networks_LH_DefaultB_PFCv_1 | Left_VIIIA_Post | 3.349 | 0.048 |
| 226 | 17Networks_RH_DefaultB_PFCv_1 | Left_Crus_II_Post | 3.348 | 0.048 |
| 227 | 17Networks_RH_SomMotA_8 | Right_Crus_II_Post | 3.348 | 0.048 |
| 228 | 17Networks_LH_DefaultB_PFCd_1 | Vermis_VII | 3.348 | 0.048 |
| 229 | 17Networks_LH_DefaultB_PFCd_1 | Left_IX_Post | 3.346 | 0.048 |
| 230 | 17Networks_LH_ContB_IPL_2 | Vermis_VII | 3.344 | 0.048 |
| 231 | Right_V_Ant | Right_VIIB_Post | 3.344 | 0.048 |
| 232 | 17Networks_RH_DefaultB_Temp_1 | Left_Crus_II_Post | 3.343 | 0.048 |
| 233 | 17Networks_RH_DefaultB_PFCd_1 | Vermis_IX | 3.342 | 0.048 |
| 234 | 17Networks_LH_SomMotB_Cent_1 | Left_VIIIB_Post | 3.342 | 0.048 |
| 235 | 17Networks_RH_TempPar_1 | Vermis_IX | 3.342 | 0.048 |
| 236 | 17Networks_LH_SomMotA_13 | Left_Crus_I_Post | 3.341 | 0.048 |
| 237 | 17Networks_LH_DefaultB_PFCd_2 | Vermis_X | 3.339 | 0.048 |
| 238 | Left_Crus_II_Post | Vermis_VIII | 3.339 | 0.048 |
| 239 | 17Networks_LH_ContB_PFClv_3 | Left_VIIIB_Post | 3.338 | 0.048 |
| 240 | 17Networks_LH_SomMotB_Aud_2 | Left_Crus_II_Post | 3.337 | 0.048 |
| 241 | 17Networks_LH_ContB_IPL_1 | Right_X | 3.337 | 0.048 |
| 242 | 17Networks_LH_ContA_PFClv_1 | Right_VI_Post | 3.336 | 0.048 |
| 243 | Right-Caudate | Vermis_VII | 3.334 | 0.048 |
| 244 | 17Networks_LH_DefaultB_PFCv_4 | Left_Crus_II_Post | 3.334 | 0.048 |
| 245 | 17Networks_LH_DefaultB_PFCv_2 | Left_VIIB_Post | 3.334 | 0.048 |
| 246 | 17Networks_RH_DefaultB_Temp_1 | Right_X | 3.328 | 0.048 |
| 247 | 17Networks_RH_VisCent_ExStr_10 | Left_Crus_II_Post | 3.325 | 0.048 |
| 248 | 17Networks_LH_DefaultB_PFCv_5 | Left_IX_Post | 3.324 | 0.048 |
| 249 | 17Networks_LH_DefaultB_PFCd_2 | Left_VIIIB_Post | 3.324 | 0.048 |
| 250 | 17Networks_LH_SalVentAttnA_Ins_1 | Left_VIIIB_Post | 3.323 | 0.048 |
| 251 | 17Networks_RH_DorsAttnB_TempOcc_1 | Right_VIIB_Post | 3.323 | 0.048 |
| 252 | Right-Amygdala | Vermis_VIII | 3.321 | 0.048 |
| 253 | 17Networks_RH_SomMotA_12 | Left_VIIB_Post | 3.319 | 0.049 |
| 254 | 17Networks_LH_VisCent_ExStr_7 | Left_Crus_II_Post | 3.319 | 0.048 |
| 255 | 17Networks_LH_ContB_IPL_1 | Right_VIIB_Post | 3.316 | 0.049 |
| 256 | 17Networks_RH_DefaultB_PFCd_3 | Left_VI_Post | 3.316 | 0.049 |
| 257 | 17Networks_LH_ContB_IPL_2 | Vermis_VIII | 3.315 | 0.049 |
| 258 | 17Networks_RH_ContB_IPL_2 | Right_VIIIA_Post | 3.313 | 0.049 |
| 259 | 17Networks_RH_DefaultA_PFCm_4 | Left_Crus_II_Post | 3.313 | 0.049 |
| 260 | 17Networks_LH_SalVentAttnA_FrMed_3 | Right_Crus_II_Post | 3.313 | 0.049 |
| 261 | 17Networks_RH_DefaultB_PFCd_1 | Vermis_X | 3.304 | 0.049 |
| 262 | 17Networks_RH_DefaultA_PFCm_6 | Left_VIIIB_Post | 3.301 | 0.049 |
| 263 | Right-Putamen | Right_VIIIB_Post | 3.301 | 0.049 |
| 264 | 17Networks_RH_DorsAttnA_TempOcc_2 | Left_Crus_II_Post | 3.300 | 0.049 |
| 265 | 17Networks_LH_DefaultB_PFCd_3 | Right_X | 3.300 | 0.049 |
| 266 | 17Networks_LH_DefaultB_PFCv_5 | Left_Crus_II_Post | 3.299 | 0.049 |
| 267 | 17Networks_LH_DefaultB_PFCd_3 | Left_Crus_II_Post | 3.299 | 0.049 |
| 268 | 17Networks_LH_SalVentAttnA_ParMed_1 | Left_VIIB_Post | 3.298 | 0.049 |
| 269 | 17Networks_LH_ContB_IPL_1 | Right_VIIIA_Post | 3.298 | 0.049 |
| 270 | 17Networks_RH_SomMotB_Cent_2 | Right_X | 3.298 | 0.049 |
| 271 | 17Networks_LH_SalVentAttnA_Ins_1 | Left_Crus_II_Post | 3.298 | 0.049 |
| 272 | 17Networks_LH_DorsAttnB_PostC_5 | Vermis_X | 3.297 | 0.049 |
| 273 | 17Networks_LH_DefaultB_PFCd_4 | Right_X | 3.295 | 0.049 |
| 274 | 17Networks_RH_DefaultA_Temp_1 | Vermis_VIII | 3.294 | 0.049 |
| 275 | 17Networks_RH_SalVentAttnB_PFClv_1 | Left_Crus_II_Post | 3.293 | 0.049 |
| 276 | 17Networks_LH_DorsAttnA_SPL_5 | Vermis_VII | 3.292 | 0.049 |
| 277 | 17Networks_RH_ContB_PFClv_1 | Left_VIIB_Post | 3.291 | 0.049 |
| 278 | 17Networks_LH_ContA_PFCl_2 | Corpus_Medullare | 3.290 | 0.049 |
| 279 | 17Networks_LH_ContB_PFCmp_1 | Vermis_VII | 3.288 | 0.049 |
| 280 | 17Networks_RH_SalVentAttnA_FrOper_2 | Left_VIIB_Post | 3.288 | 0.049 |
| 281 | 17Networks_LH_ContA_PFCl_2 | Left_Crus_I_Post | 3.286 | 0.049 |
| 282 | 17Networks_RH_ContB_IPL_1 | Vermis_VII | 3.285 | 0.049 |
| 283 | 17Networks_RH_TempPar_1 | Left_Crus_I_Post | 3.279 | 0.049 |
| 284 | 17Networks_LH_SomMotA_7 | Right_Crus_II_Post | 3.278 | 0.049 |
| 285 | 17Networks_RH_ContA_IPS_1 | Vermis_VI | 3.278 | 0.049 |
| 286 | 17Networks_RH_DorsAttnB_TempOcc_1 | Right_VIIIA_Post | 3.277 |  |

***Table S7: Harmonization of DSM-IV and DSM-5 PTSD symptom clusters***

|  | ***DSM-5 Symptom Clusters*** | | | |
| --- | --- | --- | --- | --- |
|  | **B**  **Re-Experiencing** | **C**  **Avoidance** | **D**  **Cognition and Mood** | **E**  **Hypervigilance** |
| **CAPS-IV** |  |  |  |  |
| Items | B1-B5 | C1-C2 | C3-C7 | D1-D5 |
| # of symptoms | 5 | 2 | 5 | 5 |
| Scale | 0-8 | 0-8 | 0-8 | 0-8 |
| Max total | 40 | 16 | 40 | 40 |
| **CAPS-5** |  |  |  |  |
| Items | B1-B5 | C1-C2 | D1-D7 | E1-E6 |
| # of symptoms | 5 | 2 | 7 | 6 |
| Scale | 0-4 | 0-4 | 0-4 | 0-4 |
| Max total | 20 | 8 | 28 | 24 |
| **PCL-5** |  |  |  |  |
| Items | 1-5 | 6-7 | 8-14 | 15-20 |
| # of symptoms | 5 | 2 | 7 | 6 |
| Scale | 0-4 | 0-4 | 0-4 | 0-4 |
| Max total | 20 | 8 | 28 | 24 |
